# Metagenomic Next-Generation Sequencing of Bloodstream Microbial Cell-Free Nucleic Acid in Children With Suspected Sepsis in Pediatric Intensive Care Unit

**DOI:** 10.3389/fcimb.2021.665226

**Published:** 2021-08-24

**Authors:** Gangfeng Yan, Jing Liu, Weiming Chen, Yang Chen, Ye Cheng, Jinhao Tao, Xiaodi Cai, Yang Zhou, Yixue Wang, Mingbang Wang, Guoping Lu

**Affiliations:** ^1^Paediatric Intensive Care Unit, Children’s Hospital of Fudan University, National Children’s Medical Center, Shanghai, China; ^2^BGI PathoGenesis Pharmaceutical Technology Co., Ltd, BGI-Shenzhen, Shenzhen, China; ^3^Shanghai Key Laboratory of Birth Defects, National Health Commission (NHC) Key Laboratory of Neonatal Diseases, Division of Neonatology, Children’s Hospital of Fudan University, National Children’s Medical Center, Shanghai, China

**Keywords:** bloodstream infection, metagenomic next-generation sequencing, pediatric intensive care unit, *Pneumocystis jirovecii*, sepsis

## Abstract

Bloodstream infection is a life-threatening complication in critically ill patients. Multi-drug resistant bacteria or fungi may increase the risk of invasive infections in hospitalized children and are difficult to treat in intensive care units. The purpose of this study was to use metagenomic next-generation sequencing (mNGS) to understand the bloodstream microbiomes of children with suspected sepsis in a pediatric intensive care unit (PICU). mNGS were performed on microbial cell-free nucleic acid from 34 children admitted to PICU, and potentially pathogenic microbes were identified. The associations of serological inflammation indicators, lymphocyte subpopulations, and other clinical phenotypes were also examined. mNGS of blood samples from children in PICU revealed potential eukaryotic microbial pathogens. The abundance of *Pneumocystis jirovecii* was positively correlated with a decrease in total white blood cell count and immunodeficiency. Hospital-acquired pneumonia patients showed a significant increase in blood bacterial species richness compared with community-acquired pneumonia children. The abundance of bloodstream bacteria was positively correlated with serum procalcitonin level. Microbial genome sequences from potential pathogens were detected in the bloodstream of children with suspected sepsis in PICU, suggesting the presence of bloodstream infections in these children.

## Introduction

Pathogenic microbes, including conditional pathogenic microbes, can invade the bloodstream, where they grow and reproduce, resulting in bloodstream infections. The clinical diagnosis of bloodstream infections mainly depends on the clinical manifestations, such as leukocytes and neutrophils in patients with acute high fever, as well as the microbiological test results (Timsit et al., 2020). The correct diagnosis of a bloodstream infection is essential for initiating antibiotic treatment in a timely manner (Bassetti et al., 2016). The abundance of microorganisms in the blood is low, the volume of blood obtained clinically is limited, and some microorganisms are difficult to culture, which will affect the positive rate of blood culture and turnaround time (TAT), and positive blood culture and TAT directly affects the effectiveness of clinical intervention (Lee et al., 2007; Lamy et al., 2016). At the same time, because of the complexity of microbial genomic sequences and the large number of unknown microbes in the bloodstream, conventional nucleic acid detection techniques, such as polymerase chain reaction (PCR) and DNA microarray technology, are unsuitable for this purpose (Tissari et al., 2010). Furthermore, 16S rRNA gene sequencing technology has been used to investigate the bacterial composition of blood (Rutanga et al., 2018). Watanabe et al. (2018) used 16S rRNA gene sequencing to analyze the pathogenic bacterial composition of the blood of six patients with sepsis and four healthy volunteers to the species level. Using the same technology, Li et al. (2018) compared the bacterial nucleic acid sequences of the blood of healthy controls and severe acute pancreatitis (SAP) patients, and found that SAP patients showed a significant increase in the abundance of Bacteroides and Pachycephalus and a significant decrease in the abundance of Actinomycetes. Taken together, these studies indicate the presence of a microbiome in the blood, and identifying its composition may provide novel insights into the characteristics and diagnosis of sepsis in patients.

Metagenomics next-generation sequencing (mNGS) technology has been used to investigate the microbiome of the bloodstream. Thousands of pathogens are known to infect humans, but only a fraction of them can be identified using current clinical microbiology methods. The newly developed mNGS technology enables the rapid diagnosis of unexplained infections. However, mNGS is subject to interference from host cells in the blood, as recent studies have shown that blood contains nucleic acid from every tissue in the human body, including microbial cell-free nucleic acid (mcfDNA) (De Vlaminck et al., 2015). A new solution has been provided by mNGS detection of microbial cell-free DNA in the bloodstream. In a pilot study, Kowarsky et al. (2017) performed large-scale mNGS of microbial cell-free DNA from 1,351 blood samples of 188 patients, and they identified hundreds of new bacteria and viruses representing previously unidentified members of the human microbiome. The study also indicated the feasibility of mNGS testing based on bloodstream microbial cell-free DNA samples.

As mNGS technology progresses, it is expected to solve the challenges involved in fighting global infectious diseases by facilitating the non-culture detection of microbial pathogens. In particular, unbiased mNGS technology can quickly detect pathogens in rare or complex cases where traditional clinical microbial diagnostic methods fail (Wilson et al., 2014; Wilson et al., 2015; Cheng et al., 2019; Deng et al., 2020). Overall, the mNGS method can supplement the traditional clinical microbiology method. In the present study, we performed a retrospective mNGS analysis of blood samples of children admitted to the intensive care unit with potential sepsis; we conducted a comprehensive microbiome analysis of the blood samples to identify potential pathogens associated with clinical symptoms.

## Methods

### Participants

Children who were admitted to the Pediatric Intensive Care Unit (PICU) of the Children’s Hospital of Fudan University with suspected sepsis from February 2018 to February 2020 were recruited. The inclusion criterium was patients diagnosed with systemic inflammatory response syndrome (SIRS) with reference to the international sepsis diagnostic criteria (Goldstein et al., 2005). The exclusion criteria were the following: patient age <28 days or >18 years; non-infectious causes of fever, such as connective tissue disease and central hyperthermia; patients with contraindications for fiberoptic bronchoscopy; patients who died within 24 hours of PICU or gave up treatment when the diagnosis was not yet clear. The study was approved by the Ethics Committee of the Children’s Hospital of Fudan University and is in line with the Declaration of Helsinki.

### Serum Inflammation Markers

Two milliliters of venous blood were drawn and stored at room temperature with heparin anticoagulant. The serum lipopolysaccharide (LPS) concentration was detected using the LKLM Kinetic Tube Reader (Lab Kinetics, Hutto, Texas, USA) with reference to the manufacturer’s instructions; serum procalcitonin (PCT) and interleukin-6 (IL-6) concentrations were measured by Cobas e 602 electrochemiluminescence analyzer (Roche, Basel, Switzerland), and serum C-reactive protein (CRP) concentrations were determined by QuikRead go CRP analyzer (Orion Diagnostica Oy, Finland).

### Lymphocyte Subpopulation

Using the 2ml whole blood samples, Lymphoprep Separation Medium (ready-to-use, Axis-Shield, Norway) was first used to separate peripheral blood mononuclear cells with reference to the manufacturer’s instructions. Then BD Biosciences’ fluorescently labeled monoclonal antibodies were used to label the different subsets of peripheral blood lymphocytes, i.e., anti-CD19 antibody to label B cells, anti-CD3 and anti-CD8 antibodies to label CD8+ T cells, anti-CD3 and anti-CD4 antibodies to label CD4+ T cells, and anti-CD16 and anti-CD56 antibodies to label NK cells. Finally, quantitative analysis of the lymphocyte subpopulation was performed on a flow cytometer (BD Biosciences, San Jose, CA).

### Harvest Microbial Cell-Free DNA

First, 3-4 ml whole blood samples were centrifuged at 1600 × *g* for 15 minutes to harvest the upper plasma sample. Then, using a 0.2-ml aliquot of the harvested plasma sample, we extracted microbial cell-free DNA using the TIANamp Micro DNA Kit (Catalog No. DP316, TIANGEN Biotech, Beijing, China) according to the manufacturer’s instructions, Finally, the obtained microbial cell-free DNA was frozen at −80°C for subsequent mNGS library construction.

### Harvesting of Microbial Cell-Free RNA

Using 0.2 ml of the patient’s plasma samples, the QIAamp ViralRNA Mini Kit (Cat. No. 52904, QIAGEN, Germany) was used to harvest microbial cell-free RNA, following the manufacturer’s instructions. Then, we reverse-transcribed RNA to cDNA with RNA Super Script II reverse transcriptase (Thermo Fischer Technology, Waltham, Massachusetts, USA). Finally, double-stranded DNA was further synthesized under the action of DNA polymerase I (Cat. No. P7050L, Enzymatics, USA), and the double-stranded DNA was frozen at −80°C for subsequent mNGS library construction.

### Library Construction

First, the MGIEasy Cell-free DNA Library Prep Kit (MGI Technology, Shenzhen, China) was used to construct the library according to the manufacturer’s instructions, including DNA fragment end repair, adaptor linking, and PCR amplification. Then a 2100 Bioanalyzer (Agilent, USA) was used for library quality control, and the qualified library fragment size was 200-300 bp. We used the Qubit dsDNA HS Assay Kit (Thermo Fisher Scientific) to measure the DNA library concentration, and the qualified library concentration was greater than 2 ng/μL. Finally, different samples were mixed to provide the same amount of nucleic acid for subsequent operations.

### High-Throughput Sequencing

High-throughput sequencing was performed using BGISEQ-50 (MGI Technology, Shenzhen, China), according to the manufacturer’s manual. Brief methods are as follows: first, the library was thermally denatured to form single-stranded DNA, which was circularized to form a single-stranded circular structure, which was then amplified using rolling circle amplification technology to form a DNA Nano Ball (DNB, DNA Nano Ball); finally, we completed high-throughput sequencing in the single-end 50 bp sequencing mode.

### Sequencing Data Preprocessing

The quality control work of removing the host and low-quality sequences from the metagenomic data is completed by referring to the previously published studies (Wang et al., 2019a; Wang et al., 2019b; Zhou et al., 2019; Xu et al., 2020), in brief: First, for data quality control, we removed the low-quality reads with sequence lengths of less than 35 bp using Trimmomatic software (version 0.39) (Bolger et al., 2014); then BWA software (version 0.7.15-r1140) (Li and Durbin, 2009) was used to align the high-quality reads to the human reference genome (version hg19) to remove contamination by human sequences. Duplicate sequences and low complexity sequences were removed with Prinseq software (version 0.20.4) (Schmieder and Edwards, 2011). Then, the remaining clean reads was blasted to BGI’s internal pathogen database, including 4945 viruses and 6039 bacteria (excluding Mycobacteria) related to human diseases, 174 species of mycobacterium, 137 species of mycoplasma, 1064 species of fungus, and 234 species of protozoa. The principle of reporting positive in the Central Laboratory of BGI is: a. The number of strictly aligned sequences for bacteria(except for Mycobacterium tuberculosis),fungi, and viruses is greater than 3; b. The number of strictly aligned sequences for parasites is greater than 100; C.Strictly aligned sequence book of Mycobacterium tuberculosis is greater than 1. Finally, the microbial abundance results were interpreted as viral, bacterial, fungal, or protozoan pathogens.

### Microbial Ecological Diversity

First, clean reads for each sample were normalized according to the number of reads per million (rpm) of total sequencing reads. Then, we computed the microbial ecological diversity index of each sample, including species abundance, species count, Chao1 diversity index, and Shannon diversity index, using the vegan package of R software (version 3.6.1).

### Pathogen Identification

The method of Zinter et al. (2019) was used to determine the potential pathogens in the sample, but slight modifications were made. The details are as follows: the rpm value of each detected microbe was calculated for each sample; then, each rpm value was normalized according to log10 (rpm). The Z-score of the detected microbe for each sample was defined using the following equation:

sample log10 (rpm)-mean value of all other samples log10 (rpm)/standard deviation of all other samples lo9g10 (rpm)

Finally, the possible pathogenic microbes were identified according to rpm ≥ 10 and Z-score ≥ 2.

### Correlation/Regression Analysis

To begin, the Pearson’s correlation coefficient of each microbial ecological diversity index and clinical phenotype was calculated using the cor.test function in R software. The lm function was then used to linearly fit the microbial ecological diversity index and the clinical phenotype, and a scatter plot was further drawn using the ggplot2 package. Finally, we performed the Wilcoxon rank-sum test for statistical significance assessment using the wilcox.test function.

## Results

A total of 34 children admitted to the PICU with suspected sepsis were included in this study; there were 21 males and 13 females aged 3.9 ± 3.8 years, and the final 18 cases were diagnosed as having sepsis. Through an in-depth clinical evaluation, we found that a total of 18 children could be diagnosed with pneumonia, seven of which had community-acquired pneumonia (CAP) and six had hospital-acquired pneumonia (HAP). The clinical information statistics are shown in **Table 1**.

**Table 1:** Statistics of patients’ clinical information.

Item	Summary n (%) or average ± SD
Sex	Female=13 (38.2%) male=21 (61.8%)
Age (years)	3.9 ± 3.8
Sepsis	18 (53%)
Septic shock	4(12.8%)
Pneumonia	CAP=7 (20.6%), HAP=6 (17.6%), Unknown=21 (61.8%)
Clinical microbiology	no=20 (58.8%), yes=14 (41.2%)
Oxygenation index	5.2 ± 6
Respiratory failure	12 (35.3%)
WBC (×10^9^/L)	17 ± 16
CD3+ (×10^6^/L)	1900 ± 4600
CD4+ (×10^6^/L)	770 ± 1100
CD56+ (×10^6^/L)	130 ± 140
CD8+ (×10^6^/L)	1100 ± 3600
CRP (mg/L)	74 ± 59
PCT (ng/ml)	20 ± 26
IL-6 (pg/ml)	700 ± 1100
LPS (EU/ml)	0.13 ± 0.33
Temperature (℃)	39 ± 1.4
Heart rate (BPM)	150 ± 21
hospital stay (days)	44 ± 35
Mortality (ab,yes,no,)	ab=6 (17.6%), no=22 (64.7%), yes=5 (17.7%)
PICU stay (days)	24 ± 20
Weight (kg)	16 ± 11
immunodeficiency	6 (17.6%)
post transplantation	8 (23.5%)

BPM, beat per minute; CRP, C-reactive protein; HAP, hospital-acquired pneumonia; IL-6, Interleukin-6; LPS, Lipopolysaccharide; Sd, standard deviation; PCT, procalcitonin; WBC, white blood cell; ab, abandoned.

### Bloodstream Microbiota Diversity of Children in PICU

**Figure 1:**
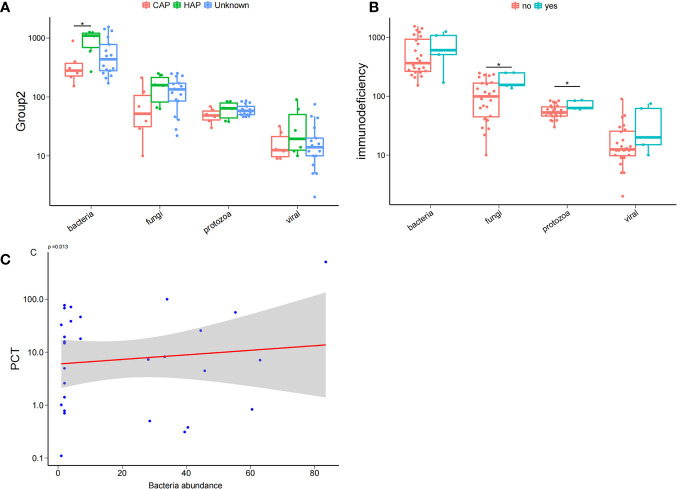
Bloodstream microbial ecological diversity in children in PICU. **(A)** The number of bloodstream bacterial species in children with hospital-acquired pneumonia (HAP) was significantly higher than in those with community-acquired pneumonia (CAP), P < 0.05, Wilcoxon rank-sum test. **(B)** The number of bloodstream fungi and protozoa in children with immunodeficiency disease was significantly higher compared with non-deficient children, P < 0.05, Wilcoxon rank-sum test; **(C)** PCT was positively correlated with the bacteria abundance. *P < 0.05.

To comprehend the composition of bloodstream microbiota in the PICU, we conducted shotgun metagenomic sequencing of microbial cell-free DNA/RNA from blood samples and obtained 42 ± 19 million sequencing reads for each sample. From the species-level bloodstream microbiota compositional data, the microbial ecological diversity was computed and compared between blood samples from patients with different clinical phenotypes. We found that, compared with children with CAP, the number of bloodstream bacterial species in those with HAP were significantly increased. Similarly, we found that the number of bloodstream fungal and protozoa species in children with immunodeficiency disease was significantly increased compared with children with non-immune deficiency disease. It is worth noting that the serum inflammation indicator PCT was significantly and positively correlated with the abundance of bacteria in the bloodstream, as shown in **Figure 1**.

### Potential Pathogenic Bacteria in Bloodstream of Children in PICU

**Figure 2:**
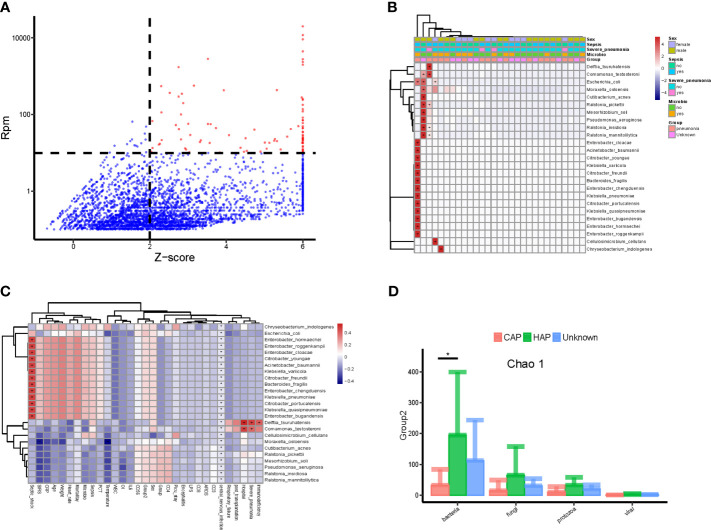
Potential pathogenic bacteria in the bloodstream of children with suspected sepsis in PICU. **(A)** Identified bloodstream pathogenic bacteria, where red dots represent potential pathogenic bacteria, Z-score > 6, uniformly assigned value was Z-score = 6. **(B)** Heat map of bloodstream potential pathogenic bacteria; each line on the horizontal represents a sample and lines on the vertical represent potential pathogenic bacteria; the “+” and “-” signs represent significant positive and negative correlations, respectively; the top is the clinical phenotype, and the color block on the right represents the specific value of clinical phenotype. **(C)** The correlation between bloodstream pathogenic bacteria and clinical phenotype; each line on the horizontal represents a clinical phenotype and on the vertical represents potential pathogenic bacteria; the “+” and “-” represent significant positive and negative correlations, respectively; the color block on the right represents the value of the correlation coefficient: positive correlation is red and negative correlation is blue. **(D)** The species richness (chao 1) of hospital-acquired pneumonia (HAP) bacteria was significantly higher than that of community-acquired pneumonia (CAP), P < 0.05, Wilcoxon rank- sum test. *P < 0.05.

The method of Zinter *et al.* (Zinter et al., 2019) was applied to identify the potential pathogenic microorganisms. As shown in **Figure 2A**, the pathogenic microbes accounted for only a small part of all detected microbes and included *Escherichia coli*, *Pseudomonas aeruginosa*, *Acinetobacter baumannii*, and *Klebsiella pneumoniae*. Correlation analyses of the abundance (RPM value) of candidate pathogenic bacteria and important clinical phenotypes, including sex, sepsis, severe pneumonia, and positive microbial culture, were conducted. As shown in **Figure 2B**, candidate pathogenic bacteria that were significantly correlated with important clinical phenotypes were found in a few (5/34) samples. While the clinical phenotypes with significant correlations with all the candidate pathogenic bacteria also showed a certain degree of aggregation. Interestingly, nearly half of the candidate pathogenic bacteria were positively correlated with septic shock (**Figure 2C**). Furthermore, we found that the value range of bacteria richness(Chao1) is 50-400,and the children with HAP had significantly higher bloodstream bacteria (Chao 1) than those with CAP **(** **Figure 2D** **)**.

### Potentially Pathogenic Viruses in the Bloodstream of Children in PICU

**Figure 3:**
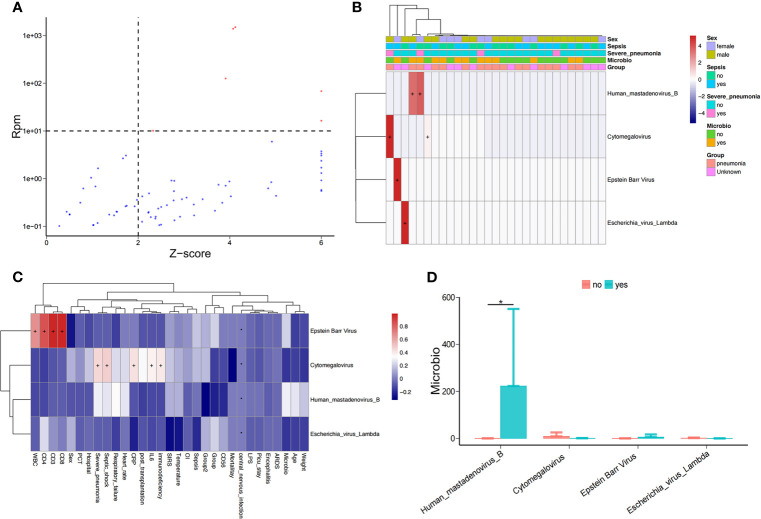
Potential pathogenic viruses in the bloodstream of children with suspected sepsis in PICU. **(A)** Identified bloodstream potential pathogenic viruses. **(B)** Heat map of bloodstream potential pathogenic viruses. **(C)** Heat map of the correlation between clinical phenotypes and candidate viruses. **(D)** Compared with children with negative microbial culture, the bloodstream abundance of *human mastadenovirus B* in children with positive microbial culture was significantly increased, P < 0.05, Wilcoxon rank-sum test. *P < 0.05.

As above, we detected the nucleic acid sequences of potentially pathogenic viruses in the bloodstream of PICU patients (**Figure 3A**), including *human mastadenovirus B*, cytomegalovirus(CMV), and Epstein-Barr virus(EBV); however, only a small number of samples (5/34) had viral sequences (**Figure 3B**). We found that the abundance of EBV in children in the PICU was positively correlated with their lymphocyte subpopulations, including total white blood cell count (WBC), CD4+ T cell count, CD3+ T cell count, and CD8+ T cell count (**Figure 3C** and **Supplementary Figure 1**), but the virus was significantly and positively correlated with serum inflammation indicators, including CRP and IL-6 levels. Of note, the abundance of serum CMV in children in the PICU was positively correlated with septic shock and immunodeficiency (**Figure 3C**), and the abundance of *human mastadenovirus B* in the blood of children with positive microbial cultures was significantly higher compared with children with negative microbial cultures (**Figure 3D**).

### Potential Eukaryotic Pathogens in Bloodstream of Children in PICU

**Figure 4:**
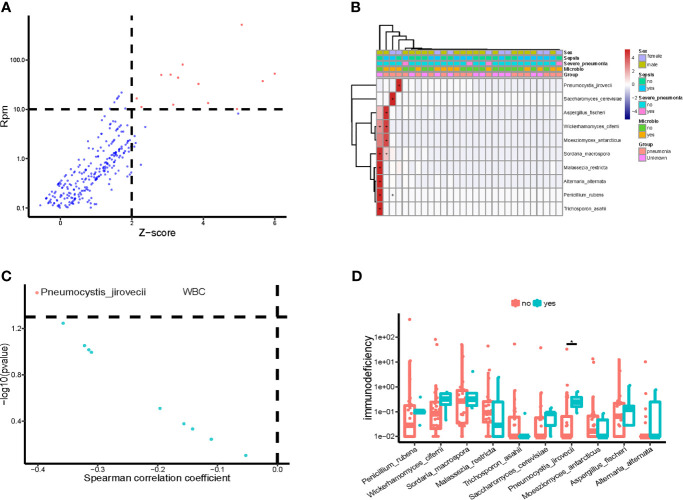
Potential pathogenic fungi in blood of children in PICU. **(A)** Identified bloodstream pathogenic fungi. **(B)** Heatmap of bloodstream pathogenic fungi. **(C)** Correlation of potential pathogenic fungi and white blood cell count (WBC). **(D)** Compared with non-immune deficient children, the bloodstream abundance of *Pneumocystis jirovecii* was significantly higher in children with immunodeficiency disease, P < 0.05, Wilcoxon rank-sum test. *P < 0.05.

We also identified the potential eukaryotic microbes in the bloodstream of children in PICU, and detected a total of 19 potential pathogenic fungi (**Figure 4A**), including *Pneumocystis jirovecii*, *Saccharomyces cerevisiae*, *Aspergillus fischeri*, *Wickerhamomyces ciferri*, *Moesziomyces antarcticus*, *Sordaria macrospora*, *Malassezia restricta*, *Alternaria alternata*, *Penicillium rubens*, and *Trichosporon asahii*. Similar to the pathogenic bacteria detected, we found that the potential pathogenic fungi were only present in a small number (12/34) of samples (**Figure 4B**), and it is worth noting that the abundance of *P. jirovecii* in blood was significantly and negatively correlated with WBC counts (**Figure 4C**). At the same time, we found a significantly higher abundance of *P. jirovecii* in the blood of immunodeficient children than in the blood of those who were non-immunodeficient (**Figure 4D**).

We investigated the correlation between important clinical phenotypes in PICU and bloodstream pathogenic fungi, and found that *P. jirovecii* was significantly more abundant in the bloodstream of children who gave up treatment than in that of non-death children (**Supplementary Figure 2A**), and those patients who received the transplantation treatment had significantly higher abundances of bloodstream *P. jirovecii* than those who did not (**Supplementary Figure 2B**). In addition, compared with children with CAP, the abundances of *A. fischeri*, *T. asahii*, and *P. rubens* were significantly higher in the blood of children with HAP (**Supplementary Figure 2C**). Notably, the bloodstream abundances of *P. jirovecii* and *A. fischeri* were significantly and positively correlated with the occurrence of acute respiratory distress syndrome (ARDS) and septic shock, respectively, in PICU children (**Supplementary Figure 2D**).

We identified protozoan nucleic acid sequences in the blood samples from children in PICU (**Supplementary Figure 3A**). The identified protozoans included *Plasmopara halstedii*, *Leishmania mexicana*, *Nannochloropsis gaditana*, and *Eimeria acervulina*, The nucleic acid sequences of *Entamoeba dispar*, *Babesia ovata*, and *Babesia bigemina* were detected in only a few (4/34) samples (**Supplementary Figure 3B**), the situation is similar to bacterial/fungal/viral pathogens, we only found pathogenic microorganisms in a few samples. We found that the bloodstream protozoan richness (Chao 1) of immune-deficient children was significantly higher than that of non-immune-deficiency children (**Supplementary Figure 3C**).

## Discussion

### mNGS Identified the Presence of Potential Pathogenic *Pneumocystis jirovecii* in the Bloodstream of Children in PICU

Although mNGS of mcfDNA is expected to identify a wide range of potential pathogenic infections in the bloodstream, the technique has faced significant challenges during clinical application. Blauwkamp et al. (2019) detected the blood mcfDNA compositions of 350 patients with suspected sepsis using mNGS and compared these with the results of conventional clinical microbiological methods; they found that 93.7% of the mNGS results were consistent with the blood culture results. Compared with conventional microbiological methods, mNGS identified more independent causes for sepsis alarms. Furthermore, mNGS detected mcfDNA in 62 of the 166 specimens for which the cause of sepsis was not identified by conventional microbiological methods, although the mcfDNA may have originated from symbiotic organisms or microbes unrelated to sepsis alarms. In addition, mNGS should be able to detect eukaryotic pathogens of unexplained fevers. Ramesh et al. (2019) performed mNGS on blood, nasopharyngeal, and fecal samples of 94 children(2-54 months old) with high fevers from Tororo District Hospital, Uganda, Africa, and they found the most common pathogen in the blood to be *Plasmodium falciparum*, which was present in 51.1% of the samples. It is worth noting that this differs from the most common pathogen in human nasopharyngeal swabs (human rhinovirus A and C, accounting for 40%) and the most common pathogen in stool (rotavirus A, accounting for 50% of patients with diarrhea).

Metagenomic sequencing of bloodstream mcfDNA can be used to detect fungal pathogens (Armstrong et al., 2019). In this study, we detected the nucleic acid sequences of the potential pathogenic fungus *P. jirovecii* in the blood of children in PICU. *P. jirovecii* is the pathogen of pneumocystis pneumonia, which can cause a severe lung disease called *Pneumocystis jirovecii* pneumonia (Rabodonirina et al., 2013; Korkmaz Ekren et al., 2018; Le Gal et al., 2020). Interestingly, the abundance of *P. jiroveci*i in the blood of children with immunodeficiency disease was significantly higher than in those with non-immunodeficiency disease in PICU. In agreement with this, the fungal and protozoan species counts in children with immunodeficiency were significantly higher than in those with non-immune deficiency. Furthermore, the abundance of bloodstream *P. jirovecii* in children in the PICU was significantly and negatively correlated with their WBC counts. Previous studies have shown that *P. jirovecii* is one of the most common and severe opportunistic infections in immunocompromised patients, especially HIV-infected patients, transplant recipients, and patients receiving high-dose corticosteroids (Jarboui et al., 2010; White et al., 2017). Our results are consistent with previous results and suggest that PICU patients with immunodeficiency should be alert to the possibility of *P. jirovecii* infection.

### mNGS Identified the Presence of Potential Viral Pathogens in Bloodstream of Children in PICU

The identification of viral pathogens based on mNGS of blood samples remains a challenge. Anh et al. (2019) performed mNGS of 492 clinical samples, including 384 serum samples,92 nasopharyngeal swabs,10 stool samples, and 6 cerebrospinal fluid samples from 386 community-acquired sepsis patients. Although the related sequences of 47 species of virus belonging to 21 familied were found in 93% of the patients, only 13.4% of the viruses detected were known to cause human infection. The study could not directly attribute the cause of sepsis to the identified virus; however, the detection of abundant viral sequences in the bloodstream highlights the fact that viruses rapidly affect critically ill patients, especially community-acquired sepsis patients.

With the widespread use of molecular diagnostic methods, viral pathogens in patients with severe respiratory diseases have received increasing attention. Advances in next-generation sequencing technology have enabled the rapid identification of non-cultured pathogens. The currently known viruses that can cause severe respiratory viral infections include Influenza A Virus, Influenza B Virus, Rhinovirus, Enterovirus, and Respiratory Syncytial Virus, as well as the recently identified coronavirus that causes new coronavirus pneumonia (COVID-19). Moreover, the rapid identification of pathogens and the use of evidence-based supportive treatment are effective for managing severe respiratory virus infections (Emonet et al., 2017; Arabi et al., 2020).

EBV is a double-stranded-DNA virus belonging to the herpesvirus family that specifically infects humans and certain primate B cells. EBV can cause latent infection: when an infected individual develops an immunosuppressive state, it is reactivated, which can lead to infectious mononucleosis, lymphoma, and lymphoproliferative diseases. EBV reactivation is very common after kidney transplantation and is associated with increased morbidity and mortality (Cohen, 2000; Blazquez-Navarro et al., 2018; de Mel et al., 2018). In the present study, we found that the abundance of bloodstream EBV in children in PICU was positively correlated with lymphocyte subtype counts, including WBC, CD4+ T cells, CD3+ T cells, and CD8+ T cells. At the same time, we also found that the abundance of bloodstream EBV was significantly and positively correlated with serum inflammation indicators, including CRP and IL6 levels, suggesting that the activation of the immune system in PICU children may be closely related to the presence of bloodstream EBV.

CMV is one of the most pathogenic viruses in humans. Studies have shown that after the initial infection, CMV survives as a latent infection in the host and is usually mild or completely asymptomatic in patients with normal immune function; however, when immunity is reduced, CMV can escape the suppression of the immune system and lead to potentially life-threatening viremia and antigenemia (Korkmaz Ekren et al., 2018; Schildermans and De Vlieger, 2020). Consistent with the results of previous studies, we detected the nucleic acid sequences of CMV in the bloodstream of PICU patients and found the abundance of CMV in the blood of children in PICU was positively correlated with the occurrence of septic shock and immunodeficiency. Considering that the species count of bloodstream viruses was significantly and positively correlated with the serum inflammation marker PCT, there may be CMV bloodstream infections in children in the PICU that are related to changes in their immune status.

### mNGS Identified Cell-Free DNA From Bacterial Pathogens in the Bloodstream of Children in PICU

Bloodstream bacterial pathogen infection is a life-threatening complication of critically ill patients (Xu et al., 2017; Li et al., 2020), and the emergence of multi-drug resistant gram-negative bacteria in hospitalized children may increase the risk of invasive infections, which are difficult to treat in the intensive care unit (Khan et al., 2019; Labi et al., 2020). We detected the presence of cell-free DNA from bacterial pathogens, including *E. coli*, *P. aeruginosa*, *A. baumannii*, and *K. pneumoniae* in the blood of children in PICU, suggesting bloodstream infection by bacterial pathogens. Furthermore, we found that the abundance of pathogenic bacteria was positively correlated with the occurrence of septic shock, suggesting that the bacterial invasion of the bloodstream of children in PICU may be related to sepsis. Notably, children with HAP had a significantly higher bloodstream bacterial richness (Chao 1) than those with CAP, which indicates that the potential pathogenic bacteria in the blood of PICU patients were more likely to come from the hospital than the community.

This study is somewhat innovative. By performing mNGS of bloodstream microbial free DNA/RNA, we aimed to identify the difficult-to-cultivate microbes that may invade the bloodstream of children in PICU. Compared with traditional clinical microbiological methods, mNGS technology can not only detect infection by prokaryotic microorganisms, such as bacteria and viruses, but also infections by eukaryotic organisms, such as fungi and parasites. The metagenomic analysis and clinical association analysis of microbes laid the foundation for further targeting of clinical phenotype-related pathogens. However, this study also had certain limitations. We found that the presence of cell-free DNA/RNA of microbes in the blood did not necessarily mean that there were living microorganisms in the bloodstream. In future, further validation is needed, such as in-depth verification of gene expression and antibody levels. In addition, the sample size of this study was limited; in the next stage of research, we propose to enlarge the sample size to further determine the pathogens related to specific clinical subphenotypes, such as immunodeficiency, pneumonia, and death. In conclusion, we detected microbial cell-free nucleic acid sequences from potential pathogens in the blood of children with suspected sepsis in PICU, indicating the potential bloodstream infection of these patients.

## Data Availability Statement

The sequencing data has been deposited into a publicly accessible repository: https://db.cngb.org/search/project/CNP0001948.

## Ethics Statement

The studies involving human participants were reviewed and approved by the ethics committee of Children’s Hospital of Fudan University. Written informed consent to participate in this study was provided by the participants’ legal guardian/next of kin.

## Author Contributions

The design of the project was completed by GL and MW. YW conducted clinical evaluation. YaC and YeC completed sample collection. JT, XC, and YZ completed sequencing and bioinformatics analysis. GY, JL, and WC completed the writing of the manuscript. GL, YW, and MW revised the manuscript. All authors contributed to the article and approved the submitted version.

## Funding

Our research was supported by Science and Technology Commission of Shanghai Municipality (STCSM, Program No. 18411950700), by National Science Foundation of China (Program No. 82071733), by shanghai talent development funding (No. 2020115).

## Conflict of Interest

Author YZ was employed by company BGI PathoGenesis Pharmaceutical Technology Co., Ltd.

The remaining authors declare that the research was conducted in the absence of any commercial or financial relationships that could be construed as a potential conflict of interest.

## Publisher’s Note

All claims expressed in this article are solely those of the authors and do not necessarily represent those of their affiliated organizations, or those of the publisher, the editors and the reviewers. Any product that may be evaluated in this article, or claim that may be made by its manufacturer, is not guaranteed or endorsed by the publisher.

## Supplementary Material

Supplementary Figure 1 The abundance of *human gammaherpesvirus 4* in bloodstream of children in PICU was correlated with leukocyte subpopulation. The abundance of *human gammaherpesvirus 4 (EBV)* in bloodstream of children in PICU and total white blood cell (WBC) count **(A)**, CD3 T cell count **(B)**, CD4 T cell count **(C)**, and CD8 T cell count **(D)** were positively correlated; the X axis represents Spearman’s correlation coefficient, and the Y axis represents the negative logarithm of the P-value of the correlation between the clinical phenotype and the potential pathogen, namely −log10(p-value); the pathogenic microbes that were screened for significant differences are marked in red.

Supplementary Figure 2 Correlation analysis of bloodstream pathogenic fungi and clinical phenotypes of children in PICU. **(A)** Numbers of *Pneumocystis jirovecii* in the bloodstream of children in PICU who gave up treatment were significantly higher than in the blood of non-death children in PICU, ab, children who gave up treatment; no, non-death PICU children, yes, dead children. **(B)**, *P. jirovecii* abundance in bloodstream of children in PICU receiving transplantation was significantly higher than that of children in PICU not receiving transplantation. **(C)** bloodstream abundances of *Aspergillus fischeri*, *Trichosporon asahii*, and *Penicillium rubens* in children with hospital-acquired pneumonia (HAP) were significantly higher than in the blood of children with community-acquired pneumonia (CAP) in PICU. **(D)** the abundance of *P. jirovecii* in the bloodstream was significantly and positively correlated with the occurrence of acute respiratory distress syndrome (ARDS) in children, and the abundance of *A. fischeri* in the bloodstream was associated with septic shock.

Supplementary Figure 3 Protozoan nucleic acid sequences in the bloodstream of children in PICU. **(A)** the nucleic acid sequence of potential protozoa in the bloodstream of children in PICU. **(B)** heat map of potential protozoa in the bloodstream of children in PICU. **(C)** the richness (Chao 1) of bloodstream protozoa in children with immunodeficiency was significantly higher than in children without immunodeficiency, P < 0.05, Wilcoxon rank-sum test.
