# Supplementary material for: Metagenomic Next-Generation Sequencing of Bloodstream Microbial Cell-Free Nucleic Acid in Children With Suspected Sepsis in Pediatric Intensive Care Unit
Source: Front Cell Infect Microbiol. 2021 Aug 24;11:665226. doi: 10.3389/fcimb.2021.665226 (PMC8421769; doi:10.3389/fcimb.2021.665226)
Supplement: Supplementary Figure 1 — The abundance of human gammaherpesvirus 4 in bloodstream of children in PICU was correlated with leukocyte subpopulation. The abundance of human gammaherpesvirus 4 (EBV) in bloodstream of children in PICU and total white blood cell (WBC) count (A), CD3 T cell count (B), CD4 T cell count (C), and CD8 T cell count (D) were positively correlated; the X axis represents Spearman’s correlation coefficient, and the Y axis represents the negative logarithm of the P-value of the correlation between the clinical phenotype and the potential pathogen, namely −log10(p-value); the pathogenic microbes that were screened for significant differences are marked in red. [file DataSheet_1.docx]

**Supplementary Appendix**

**Supplementary Figures and legends**


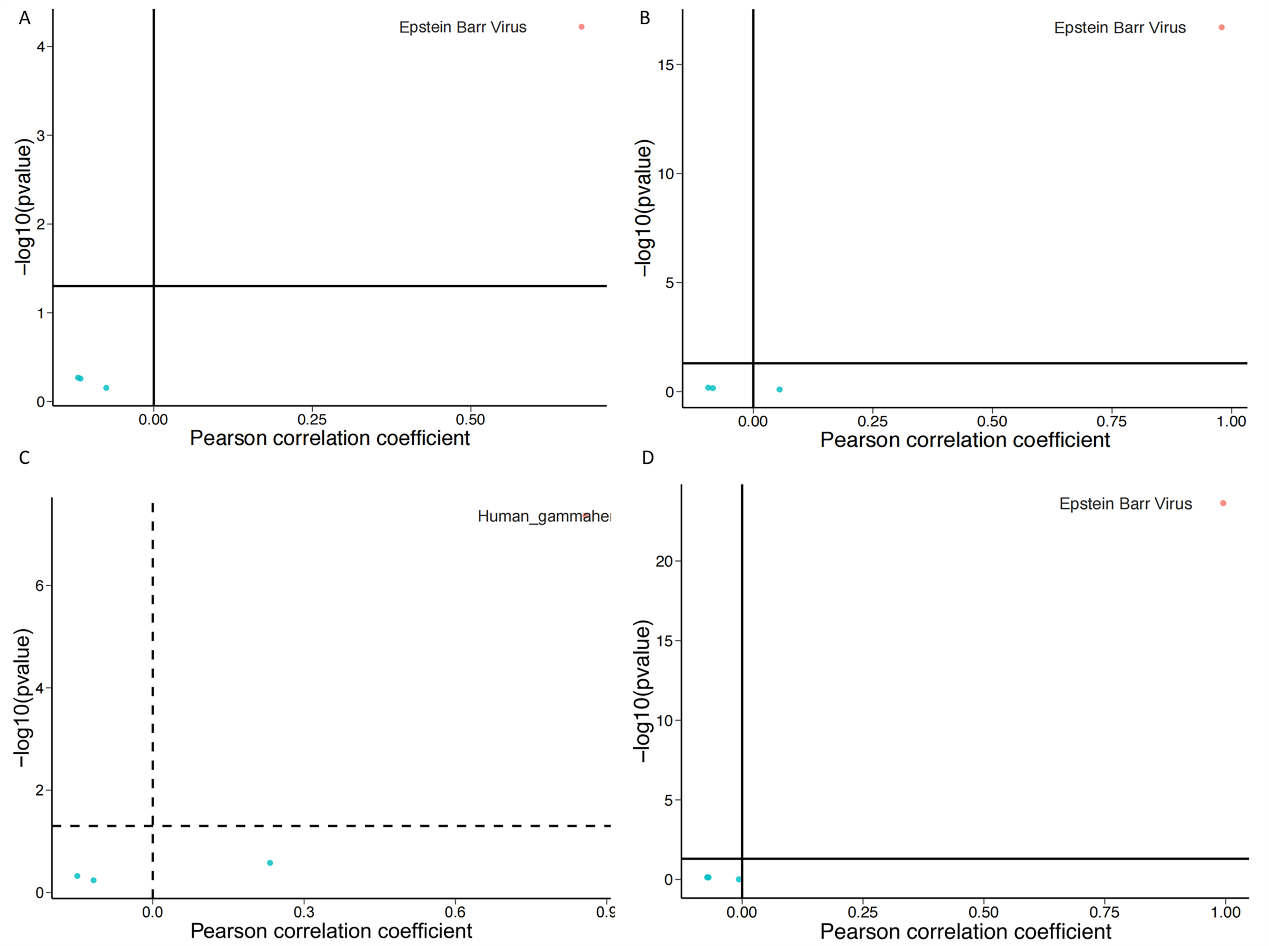


**Supplementary Figure 1. The abundance of *Epstein Barr virus* in bloodstream of children in PICU was correlated with leukocyte subpopulation.** The abundance of *EBV* in bloodstream of children in PICU and total white blood cell (WBC) count (A), CD3 T cell count (B), CD4 T cell count (C), and CD8 T cell count (D) were positively correlated; the X axis represents Spearman’s correlation coefficient, and the Y axis represents the negative logarithm of the P-value of the correlation between the clinical phenotype and the potential pathogen, namely −log10(p-value); the pathogenic microbes that were screened for significant differences are marked in red.


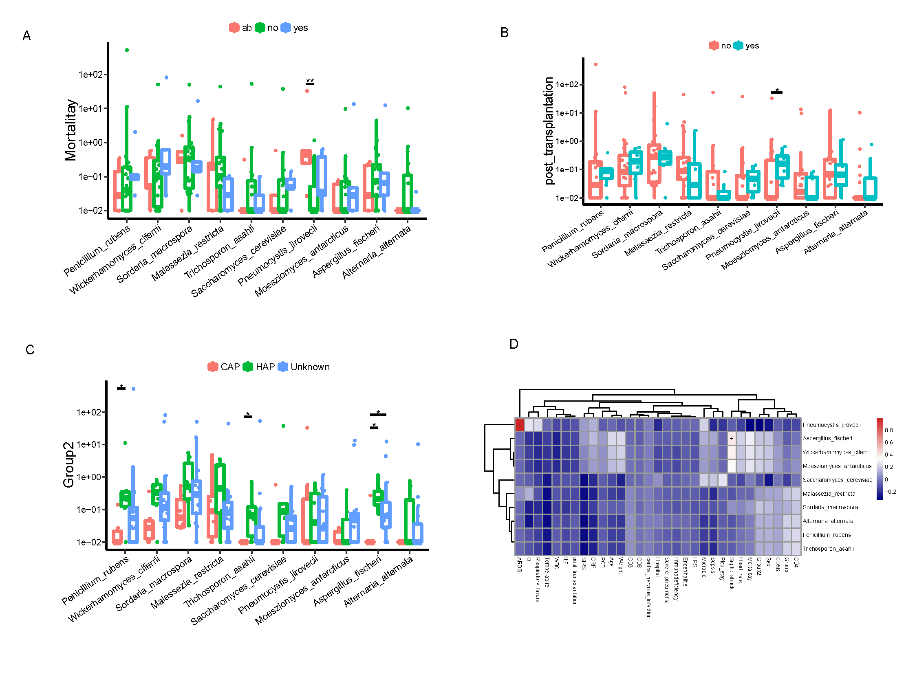


**Supplementary Figure 2. Correlation analysis of bloodstream pathogenic fungi and clinical phenotypes of children in PICU**. A, Numbers of *Pneumocystis jirovecii* in the bloodstream of children in PICU who gave up treatment were significantly higher than in the blood of non-death children in PICU, ab, children who gave up treatment; no, non-death PICU children, yes, dead children. B, *P. jirovecii* abundance in bloodstream of children in PICU receiving transplantation was significantly higher than that of children in PICU not receiving transplantation. C, bloodstream abundances of *Aspergillus fischeri*, *Trichosporon asahii,* and *Penicillium rubens* in children with hospital-acquired pneumonia (HAP) were significantly higher than in the blood of children with community-acquired pneumonia (CAP) in PICU. D, the abundance of *P. jirovecii* in the bloodstream was significantly and positively correlated with the occurrence of acute respiratory distress syndrome (ARDS) in children, and the abundance of *A. fischeri* in the bloodstream was associated with septic shock.


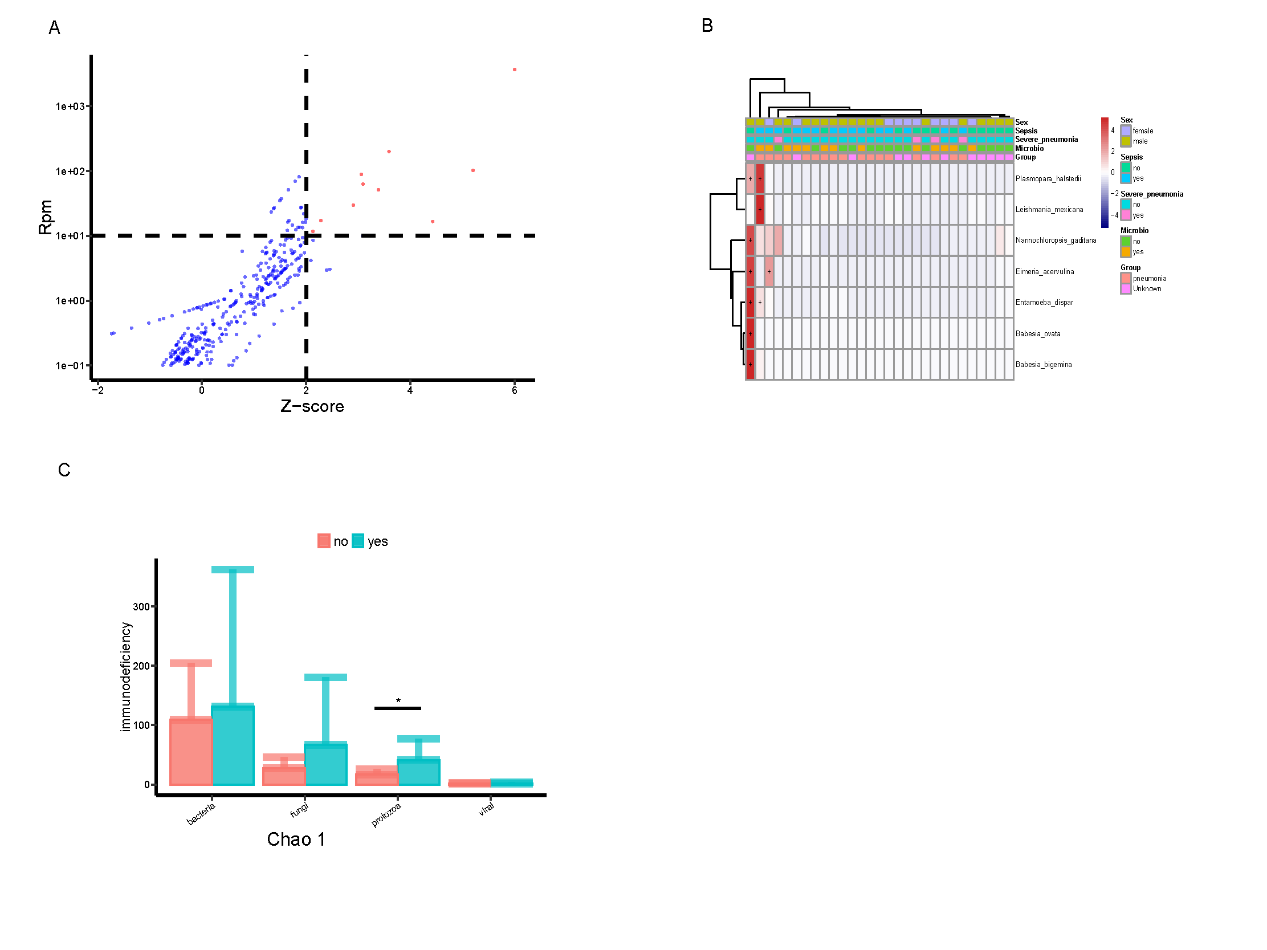


**Supplementary Figure 3**. **Protozoan nucleic acid sequences in the bloodstream of children in PICU**. A, the nucleic acid sequence of potential protozoa in the bloodstream of children in PICU. B, heat map of potential protozoa in the bloodstream of children in PICU. C, the richness (Chao 1) of bloodstream protozoa in children with immunodeficiency was significantly higher than in children without immunodeficiency, P < 0.05, Wilcoxon rank-sum test.
